# Prediction of Protein–ATP Binding Residues Based on Ensemble of Deep Convolutional Neural Networks and LightGBM Algorithm

**DOI:** 10.3390/ijms22020939

**Published:** 2021-01-19

**Authors:** Jiazhi Song, Guixia Liu, Jingqing Jiang, Ping Zhang, Yanchun Liang

**Affiliations:** 1 College of Computer Science and Technology, Jilin University, No. 2699 Qianjin Street, Changchun 130012, China; songjz671@nenu.edu.cn (J.S.); zhangping18@mails.jlu.edu.cn (P.Z.); ycliang@jlu.edu.cn (Y.L.); 2 College of Computer Science and Technology, Inner Mongolia University for Nationalities, No. 536 Huolinhe Street, Tongliao 028000, China; jiangjingqing@aliyun.com; 3 Key Laboratory of Symbolic Computation and Knowledge Engineering of Ministry of Education, Jilin University, No. 2699 Qianjin Street, Changchun 130012, China; 4 Zhuhai Laboratory of Key Laboratory of Symbolic Computation and Knowledge Engineering of Ministry of Education, Zhuhai College of Jilin University, Caotang Crescent (Wan) Jinwan District, Zhuhai 519041, China

**Keywords:** protein–ATP binding residue prediction, deep convolutional neural network, LightGBM, ensemble learning, protein primary sequence

## Abstract

Accurately identifying protein–ATP binding residues is important for protein function annotation and drug design. Previous studies have used classic machine-learning algorithms like support vector machine (SVM) and random forest to predict protein–ATP binding residues; however, as new machine-learning techniques are being developed, the prediction performance could be further improved. In this paper, an ensemble predictor that combines deep convolutional neural network and LightGBM with ensemble learning algorithm is proposed. Three subclassifiers have been developed, including a multi-incepResNet-based predictor, a multi-Xception-based predictor, and a LightGBM predictor. The final prediction result is the combination of outputs from three subclassifiers with optimized weight distribution. We examined the performance of our proposed predictor using two datasets: a classic ATP-binding benchmark dataset and a newly proposed ATP-binding dataset. Our predictor achieved area under the curve (AUC) values of 0.925 and 0.902 and Matthews Correlation Coefficient (MCC) values of 0.639 and 0.642, respectively, which are both better than other state-of-art prediction methods.

## 1. Introduction

Interactions between proteins and ligands are crucial for many biological activities and play significant roles in a wide variety of biological processes, including membrane transportation, muscle contraction, and replication and transcription of DNA [1,2,3]. Therefore, accurately identifying the positions of binding sites in proteins is instructive for protein function annotation and novel drug design for diseases like cancers [4], diabetes [5], and Alzheimer’s [6]. Adenosine-5′-triphosphate (ATP) is an important ligand molecule that serves as an energy source and a coenzyme in cell biology [7]. It interacts with proteins through protein–ATP binding residues in protein sequences and provides chemical energy to proteins via hydrolysis, which can be used for various protein functions [8,9]. Tremendous wet-lab efforts have been undertaken to identify the locations of the protein–ATP binding residues, including X-ray crystallography [10] and nuclear magnetic resonance (NMR) [11]. However, these wet-lab experiments are often cost-intensive and time-consuming [12], which constrains their application to the large-scale protein sequences of the postgenomic era. 

Given these circumstances, the use of computational approaches for protein–ATP binding residue prediction is drawing more attention from researchers as advances are made in artificial intelligence and machine learning. Depending on the protein features involved, these computational prediction methods can be grouped into two categories: sequence-based methods, in which features are derived from protein sequence information, and structure-based methods, in which features are derived from structural protein information. As of 4 November 2020, the number of protein structures in Protein Data Bank [13] (about 170,594) was relatively lower than that in the Swiss-Prot database [14] (about 563,552) because the three-dimensional structure of proteins is more difficult to detect than protein sequence information. Therefore, using the sequence information for protein–ATP binding residue prediction has wider application prospects. 

In the past two decades, numerous sequence-based methods based on machine-learning algorithms have been proposed for prediction of protein–ATP binding residues. In 2009, Chauhan et al. [15] developed ATPint, which is one of the first custom-designed computational predictors for identifying protein-ATP binding residues. With the combination of Support Vector Machines (SVMs) and Position Specific Scoring Matrix (PSSM), ATPint achieved a pioneering result, which demonstrates the practicability of computational methods for ATP-binding residues prediction. In 2011, Chen et al. [16] proposed ATPsite, which improved the predicted Area Under curve (*AUC*) value from 0.627 to 0.854. They adopted more sequence-based features including predicted protein secondary structure, and predicted solvent accessibility and residue conservation scores. Then feature selection was performed to remove irrelevant and redundant features. Finally, the selected features were fed into a SVMs classifier to generate probabilities of ATP-binding. Yu et al. [8] proposed TargetATP for protein-ATP binding residues prediction. For a protein to be predicted, the TargetATP first extracted LogisticPSSM and predicted secondary structure features of each residue and applied the sliding window technique; then, the two extracted features were combined and fed into multiple individual SVMs, the outputs of SVMs were ensembled by applying an appropriate ensemble scheme; finally, a threshold was used to determine whether the residue was an ATP-binding residue. In 2013, they developed another protein-ATP binding residue predictor named TargetATPsite [17]. The evolutionary information derived from PSSM was considered as image and further processed by sparse representation to obtain more discriminative features. To effectively deal with the imbalanced problem between the positive instances and the negative instances, multiple random under-sampling and ensemble learning were applied. TargetATPsite achieved *AUC* of 0.882 on its independent testing set, which was better than the comparative methods. In 2014, Fang et al. [18] proposed a simple method which adopted a modified PSSM encoding scheme for ATP-binding residues prediction. In their study, only the high local evolutionary conservation scores in PSSMs were considered as input, without employing any predicted features from other classifiers. Their method reached the *AUC* value of 0.899 in their dataset. The results demonstrated that PSSM plays a significant role in the protein-ATP binding mechanism. In 2018, Hu et al. [19] proposed a hybrid prediction method named ATPbind including a sequence-based predictor ATPseq and a structural-based predictor. The output of S-SITEatp, which is a sequence profile-profile comparison method, was added to the feature matrix of the ATPseq along with other sequence information. Finally, the whole feature matrix was sent into multiple SVMs classifiers which were assembled for final prediction.

Although previous studies have produced significant progress in protein–ATP binding residue prediction, with the development of artificial intelligence, some new algorithms and techniques have been proposed to improve performance in classification problems. The convolutional neural network (CNN), as one of the most important branches in the deep learning framework, has shown outstanding performance in image recognition [20,21], computer vision [22], recommendation systems [23], and natural language processing [24]. In the bioinformatics field, the CNN framework has been successfully applied in protein secondary structure prediction [25,26] and protein–ligand binding site prediction [27,28]. In 2016, Golkov et al. [29] proposed a method for predicting contact between residues in protein sequences. They turned a protein sequence into a graph-valued image and used a CNN to calculate protein contact maps from detailed evolutionary coupling statistics between positions in the protein sequence. Zhou et al. [30] proposed CNNsite, which used the CNN structure to predict DNA-binding residues in protein sequences based on sequence information. CNNsite outperforms prediction methods based on traditional machine-learning algorithms such as SVM and random forest (RF), which demonstrates the efficacy of CNN in protein–ligand binding residue prediction. In 2019, Nguyen et al. [31] applied a fine-tuned 2D CNN structure to predict ATP-binding residues in membrane proteins. After tuning the hyperparameters, the structure showed highly effective prediction performance for membrane proteins.

Compared with traditional machine-learning classification algorithms, the CNN framework automatically generates novel features by applying different sizes of convolution kernels to the input data and passing these features to the next layer, which can avoid some biases from feature engineering and reduce the mismatch between feature extraction and the classifier. To handle different classification tasks, various CNN frameworks have been constructed, such as VGG-16 [32], Inception [33], ResNet [34], and Xception [35]. Compared with the simple CNN structure, these frameworks adopt corresponding techniques to further improve the classification ability in practical applications. Therefore, applying a certain CNN framework or combining several CNN frameworks may improve the performance in protein–ATP binding residue prediction. Besides deep learning frameworks, some progress has also been achieved using other classification algorithms. In 2017, Microsoft Research Asia proposed the LightGBM [36] algorithm, which is a novel Gradient Boosting Decision Tree (GBDT) algorithm with gradient-based one-side sampling (GOSS) and exclusive feature bundling (EFB) to deal with big data and large numbers of features, respectively. Compared with traditional boosting algorithms, LightGBM shows faster training process with a lower memory cost, which makes LightGBM more suitable for high-throughput data tasks like analysis of protein sequences.

In this study, we constructed a sequence-based prediction method for protein–ATP binding residues by combining CNN frameworks and LightGBM. For the CNN frameworks, we propose a multi-incepResNet-based predictor and a multi-Xception-based predictor. Compared with traditional CNN frameworks which use the whole feature matrix as the input, we applied the corresponding convolutional neural network for each type of feature to detect deeper representations to avoid the negative effects caused by feature differences, and these representations were combined in a deeper layer of the network for classification. The outputs of the CNN frameworks and LightGBM were merged by an ensemble learning algorithm. At the same time, a complimentary template-based method was implemented to further improve the prediction performance from another angle. The final prediction consists of the ensemble learning prediction result and the complimentary template-based prediction result. In two independent testing sets, our sequence-based prediction method achieved AUC values of 0.924 and 0.902. In comparison with other existing prediction methods, our method was determined to perform significantly better in protein–ATP binding residue prediction. The full source code and benchmark datasets in this study are freely available at https://github.com/tlsjz/ATPensemble.

## 2. Results

### 2.1. Performance Comparison with other Sequence-Based Prediction Methods

To demonstrate the performance of our proposed ensemble predictor, we provide the evaluation criteria of prediction results on the classic testing dataset ATP-17 and newly proposed testing dataset ATP-41. We provide the performance of other sequence-based ATP-binding residue predictors for comparison with our proposed predictor. The evaluation criteria of other sequence-based predictors were extracted from their corresponding papers. Since most of the previous predictors were tested on the classic testing set ATP-17, their performance on the proposed testing set ATP-41 could not be obtained for some of them, namely ATPint, ATPsite, and TargetATP.

Table 1 shows the overall performance of our proposed ensemble predictor and that of other sequence-based predictors on the classic testing set ATP-17. The highest value of each column in the table is shown in bold. The classification threshold was set to 0.43, which maximized the *MCC* value when the training set was ATP-227, to calculate the *ACC*, sensitivity, specificity, and *MCC*. The *MCC* and *AUC* values of our proposed predictor were superior to those of other sequence-based predictors, with values of 0.639 and 0.925, respectively. For the other three criteria, our proposed ensemble predictor achieved a better *ACC* value at 0.978, the multi-IncepResNet-based predictor in our ensemble predictor achieved a sensitivity of 0.608, and the complementary template prediction method achieved a specificity of 0.995. However, the sensitivity of the complementary template prediction method was only 0.189. This can be explained by its very limited prediction coverage and the limited number of sequences in the training set. The *AUC* values of the multi-IncepResNet-based predictor, multi-Xception-based predictor, and the LightGBM predictor were 0.915, 0.909, and 0.917, respectively; the *MCC* values of the three subclassifiers were 0.565, 0.630, and 0.618, respectively. After applying the ensemble learning algorithm, the *AUC* and *MCC* values improved to 0.925 and 0.638, respectively, which were better than those of the subclassifiers. To further improve the accuracy for true positive instances, the complementary template prediction method was adopted, which helped to improve the *MCC* value from 0.638 to 0.639.

**Table 1:** Performance comparison between our proposed method and other sequence-based methods on ATP-17 testing set (methods are ranked according to AUC and the highest value of each column in the table is shown in bold).

Method	ACC	Sen	Spe	MCC	AUC
Complementary template	0.967	0.189	**0.995**	0.324	--
ATPint ^a^	0.665	0.512	0.660	0.066	0.606
ATPsite ^a^	0.969	0.367	0.991	0.451	0.868
NsitePred ^b^	0.967	0.460	0.985	0.476	0.875
TargetATPsite ^c^	0.972	0.458	0.991	0.530	0.882
TargetNUCs ^d^	0.975	0.516	0.992	0.584	--
Multi-Xception-based predictor	0.977	0.565	0.993	0.630	0.909
TargetATP ^a^	0.969	0.489	0.989	0.542	0.912
Multi-IncepResNet-based predictor	0.969	**0.608**	0.983	0.565	0.915
LightGBM predictor	0.977	0.556	0.992	0.618	0.917
Ensemble without template	**0.978**	0.569	0.993	0.638	**0.925**
Ensemble predictor	**0.978**	0.589	0.992	**0.639**	**0.925**

^a^: Data obtained from Reference [8]; ^b^: Data obtained from References [37,38]; ^c^: Data obtained from Reference [17]; ^d^: Data obtained from References [39,40]; --: Not available or not reported in paper.

**Figure 1:**
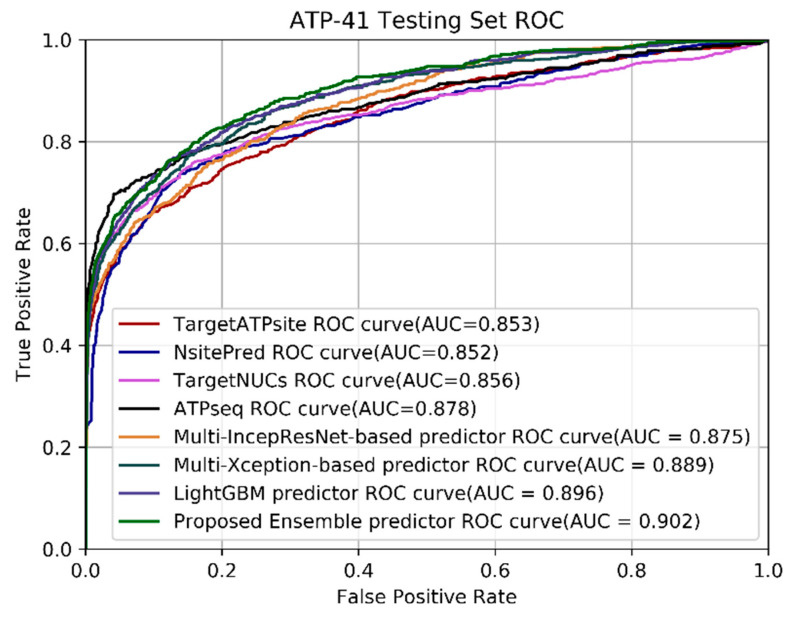
ROC curves of our proposed ensemble predictor and other sequence-based predictors on the ATP-41 testing set.

To fully verify the proposed ensemble predictor’s performance, we compared our proposed method with that of other sequence-based predictors on the newly proposed testing set ATP-41, which has more protein sequences for evaluation; the criteria values are listed in Table 2. The highest value of each column in the table is shown in bold. The classification threshold was set to 0.37 because when the training set was ATP-388, this threshold produced the highest *MCC* value. We found that the *MCC* and *AUC* of our proposed ensemble predictor are consistently better than those of the other sequence-based predictors, with values of 0.642 and 0.902, respectively. For the *ACC* criterion, our ensemble predictor achieved 0.973, which is slightly better than the other predictors. Our predictor is slightly less sensitive than ATPseq, but its specificity is lower than that of our method, which means that there could be more false positive instances in its result. For specificity, the complementary template method achieved 0.998, but its sensitivity was 0.239, which is a similar situation to that which occurred with the ATP-17 testing set. The *AUC*s of multi-IncepResNet-based predictor, multi-Xception-based predictor, and the LightGBM predictor were 0.875, 0.889, and 0.896, respectively. The *MCC*s of the multi-IncepResNet-based predictor, multi-Xception-based predictor, and the LightGBM predictor were 0.589, 0.585, and 0.597, respectively. By adopting the ensemble learning, the *AUC* and *MCC* were improved to 0.902 and 0.625 respectively; when the complementary template prediction method was used, the *MCC* of our proposed ensemble predictor was further improved to 0.642. The *ROC* curves of our proposed predictor and other sequence-based predictors on the ATP-41 testing set are shown in Figure 1, which further displays the superior generalization capability of our prediction method.

**Table 2:** Performance comparison between our proposed method and other sequence-based methods on the ATP-41 testing set (methods are ranked according to AUC and the highest value of each column in the table is shown in bold).

Method	ACC	Sen	Spe	MCC	AUC
Complementary template	0.964	0.239	**0.998**	0.451	--
NsitePred ^a^	0.954	0.467	0.977	0.456	0.852
TargetATPsite ^a^	0.968	0.413	0.995	0.559	0.853
TargetNUCs ^a^	0.972	0.469	0.997	0.627	0.856
Multi-IncepResNet-based predictor	0.969	0.441	0.995	0.589	0.875
ATPseq ^a^	0.972	**0.545**	0.993	0.639	0.878
Multi-Xception-based predictor	0.968	0.480	0.992	0.585	0.889
LightGBM predictor	0.970	0.447	0.996	0.597	0.896
Ensemble without template	0.972	0.461	0.997	0.625	**0.902**
Ensemble predictor	**0.973**	0.497	0.996	**0.642**	**0.902**

^a^: Data obtained from [19]; --: Data not available.

The outstanding performance of our proposed ensemble predictor on the two independent testing set demonstrates that the deep convolutional neural network and the LightGBM can be efficiently applied to protein–ATP binding residue prediction, and the overall prediction performance can be greatly improved by using the appropriate ensemble learning algorithm. The *MCC* of the prediction result can be further improved by combination with the complementary template prediction method.

### 2.2. Case Study

**Figure 2:**
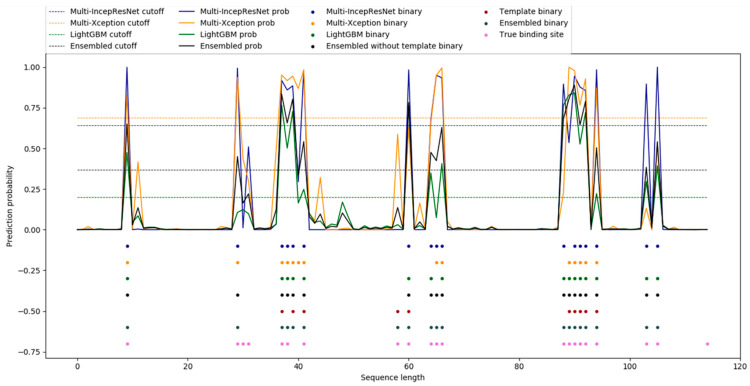
Binding residues predicted by the multi-IncepResNet-based predictor, multi-Xception-based predictor, LightGBM predictor, and the ensemble predictor for protein 4RX6_B. The *x* axis represents each residue in 4RX6_B and the *y* axis represents the probability predicted by the classifier. The blue, yellow, green, and black lines represent the probability curves of the multi-IncepResNet-based predictor, the multi-Xception-based predictor, the LightGBM predictor, and the ensemble predictor, respectively; the dots in the corresponding colors represent the binding residues predicted by the corresponding predictors. Specifically, the pink dots denote the observed (true) binding residues in the protein sequence, the red dots denote the predicted binding residues from the complementary template method, and the dots in slate-gray denote the predicted binding residues from the ensemble predictor complemented with the template method.

Next, the protein sequence in the ATP-41 testing set with the PDB ID 4RX6_B was used for a case study. The prediction results of the three subclassifiers and the ensemble predictor are shown in Figure 2.

Figure 2 shows that the multi-IncepResNet-based predictor correctly predicted 16 out of 21 true binding residues with 1 false positive instance; the multi-Xception-based predictor correctly predicted 12 out of 21 true binding residues with 2 false positive instances; the LightGBM predictor correctly predicted 15 out of 21 true binding residues with 1 false positive instance. After ensemble learning algorithm, the ensemble predictor correctly predicted 17 out of 21 true binding residues with 1 false positive instance. Finally, the performance further improved with the use of the complementary template method, with 18 correctly predicted binding residues and 1 false positive instance.

## 3. Discussions

### 3.1. Feature Importance Analysis

**Figure 3:**
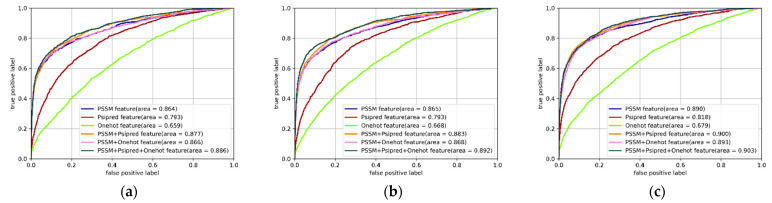
Receiver operating characteristic (*ROC*) curves of the multi-IncepResNet-based predictor (**a**), the multi-Xception-based predictor (**b**), and the LightGBM predictor (**c**) under different individual features and feature combinations. The corresponding area under the curve (*AUC*) values are listed in the legends.

In this analysis, we tried to validate the effectiveness of the included features for protein–ATP binding residue prediction. Three types of feature were applied in this study: the PSSM, the predicted secondary structure, and the one-hot encoding for each residue in the protein sequence. We compared the prediction performances of classification algorithms based on individual features and different combinations of features on the ATP-388 dataset over five-fold cross validations to demonstrate their impact on the prediction results. The *AUC* value was adopted to reveal the performance of the classifiers. The *ROC* of the multi-IncepResNet-based, multi-Xception-based, and LightGBM predictors under different features or feature combinations are shown in Figure 3a–c, respectively.

For individual features, the three classification algorithms performed better when the PSSM feature was applied, giving *AUC* values of 0.864, 0.865, and 0.890, respectively. This demonstrated that the PSSM feature, which mainly reflects the conservation of residue, is a significant property for protein–ATP binding residue prediction. For feature combinations, since the PSSM feature performed better than the other individual features in the prediction *AUC*, we set the PSSM feature as the base feature and combined other features with the PSSM. The *ROC* curves in the Figure 3 illustrate that the prediction performance with feature combination was better than the performance of individual features. The combination of PSSM and the predicted secondary structure performed better than the combination of PSSM and one-hot encoding in the three classification algorithms. When the three features were all adopted, the prediction *AUC* outperformed the other feature combinations, with 0.886, 0.892, and 0.903 for the multi-IncepResNet-based predictor, multi-Xception-based predictor, and LightGBM predictor, respectively. Therefore, by comparing the performance with individual features and feature combinations, the three included features were determined to be effective for protein–ATP binding residue prediction; of them, the PSSM feature is indispensable. When all three features were applied, the classification algorithms achieved the best performance.

### 3.2. Proposed CNN-Based Models Showed Better Performance than Simple 2D CNN Model

In this study, we constructed two CNN-based predictors: a multi-IncepResNet-based predictor and a multi-Xception-based predictor. Both predictors adopt certain CNN frameworks like Inception, ResNet, and Xception, which apply corresponding techniques to improve the classification performance. However, to show their effectiveness, we compared the prediction performance with a fine-tuned 2D CNN model on ATP-388 over five-fold cross-validations. For the structure of the fine-tuned 2D CNN model, refer to Reference [31], which aimed to predict ATP-binding residues for membrane proteins. The hyperparameters used for tuning the 2D CNN model are listed in Table 3. After hyperparameter optimization, the best set of hyperparameters was found to be 60 training epochs, a batch size of 256, learning rate of 0.001, and dropout rate of 0.4.

The prediction performance measures of the multi-IncepResNet-based predictor, multi-Xception-based predictor, and the fine-tuned 2D CNN model are listed in Table 4. The highest value in the column is shown in bold. The fine-tuned 2D CNN model achieved 0.417 and 0.871, the multi-IncepResNet-based predictor achieved 0.501 and 0.886, and the multi-Xception-based predictor achieved 0.519 and 0.892 for *MCC* and *AUC*, respectively. The *MCC*s and *AUC*s of the proposed CNN-based predictors were superior to those of the fine-tuned simple 2D CNN model because our proposed predictors apply several CNN-based frameworks, and the techniques used in these frameworks produced efficient performance improvements. For example, the Inception framework concurrently operates several convolution kernels with various sizes; this process allows the CNN to detect local features from multiple receptive fields simultaneously and concatenate these features for deeper feature extraction in the subsequent layers.

### 3.3. Applying Separate Features as Inputs in CNN Models can Improve Performance

In the common IncepResNet or Xception structures, the whole feature matrix is sent into the network as the input. This process is often effective in the fields of image representation and image recognition, where the network input is a full image. However, in protein–ATP binding residue prediction, where the network input is various sequence information features, taking all features as a whole into the network may not be the best solution.

**Figure 4:**
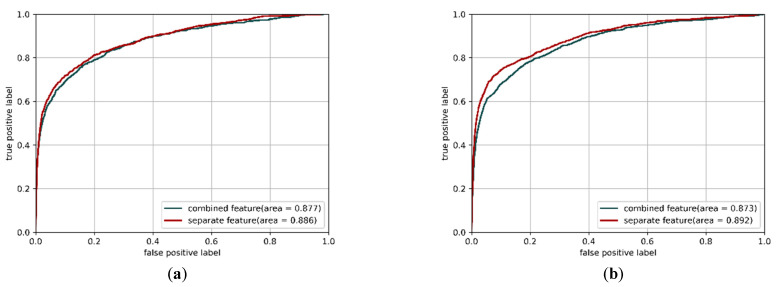
*ROC* curves of the multi-IncepResNet-based predictor (**a**) and the multi-Xception-based predictor (**b**) with combined features and separate features as the inputs. The corresponding *AUC* values are listed in the subfigure legends.

Since each sequence feature has its corresponding property for prediction, i.e., the PSSM feature represents the evolutionary conservation, the predicted secondary structure feature represents the type of protein secondary structure for the query residue, and the one-hot encoding feature represents the specific physicochemical property of the query residue, we used the separate features as inputs for the CNN structures in this study. In the multi-IncepResNet-based and multi-Xception-based predictors, three sequence features were separately fed into the network, each individual feature had the corresponding CNN structures for deep representations extraction, and the deep representations from individual features were merged for predictions. Figure 4a,b shows the prediction *ROC* using the combined feature and separate features as the inputs for the multi-IncepResNet-based predictor and the multi-Xception-based predictor, respectively, on ATP-388 over five-fold cross validation. It is worth mentioning that the hyperparameters in CNN structures with combined feature were also fine-tuned, which made the CNN structures achieve their best performance. The optimal hyperparameters for CNN structures with combined feature and CNN structures with separate features are listed in Table 5.

For the multi-IncepResNet-based predictor, using the combined feature as the input achieved an *AUC* of 0.877, while using separate feature inputs achieved an *AUC* of 0.886. For the multi-Xception-based predictor, using separate features as inputs also achieved a better *AUC* of 0.892 compared with 0.873 using the combined feature input. These improvements in the prediction *AUC* demonstrate that in protein–ATP binding residue prediction, using separate features as inputs and applying corresponding CNN structures for deep representation extraction can efficiently improve performance and reduce the negative effects of feature differences. Furthermore, the idea of using separate features as the inputs for CNN-based models can be applied to other protein prediction problems where sequence information features are adopted as features.

### 3.4. Ensemble Learning for CNN Predictors and the LightGBM Predictor

Ensemble learning can significantly improve prediction performance by combining the results from multiple subclassifiers with certain rules. Various ensemble strategies have been proposed for different types of subclassifiers, such as maximum ensemble, minimum ensemble, mean ensemble, and weighted ensemble. The definitions of the above ensemble strategies are shown as follows, respectively:

(1) $$\mu_{j}\left(x\right)=\;\underset{1\leq i\leq L}{\max}S_{i,j}\left(x\right),$$

(2) $$\mu_{j}\left(x\right)=\;\underset{1\leq i\leq L}{\min}S_{i,j}\left(x\right),$$

(3) $$\mu_{j}\left(x\right)=\;\frac{1}{L}(S_{1,j}\left(x\right)+S_{2,j}\left(x\right)+\ldots +S_{L,j}\left(x\right)$$

and

(4) $$\mu_{j}\left(x\right)=\;W_{1}*S_{1,j}\left(x\right)+W_{2}*S_{2,j}\left(x\right)+\ldots +W_{L}*S_{L,j}\left(x\right),$$

(5) $$\left(s.t.\;\sum_{i=1}^{L}W_{i}=1\right),$$

where $\mu_{j}\left(x\right)$ is the confidence of instance *x* being classified into class *j* after classifier ensemble, $S_{i,j}\left(x\right)$ is the confidence of *x* being classified into class *j* by the subclassifier *i,* and *L* is the total number of subclassifiers.

With the proper strategy, applying ensemble learning can improve the generalization performance and avoid the risk of local optimization. In this study, three subclassifiers were included: the multi-IncepResNet-based predictor, the multi-Xception-based predictor, and the LightGBM predictor. For the ensemble strategy, we tried the above four strategies on ATP-388 and ATP-227 over five-fold cross validation, and the prediction performance is reported in Table 6. The highest value in the column is shown in bold. According to Table 6, the weighted ensemble strategy achieved better *MCC* and *AUC* values on both ATP-388 and ATP-227 datasets; therefore, the weighted ensemble strategy was adopted to combine the three subclassifiers.

**Figure 5:**
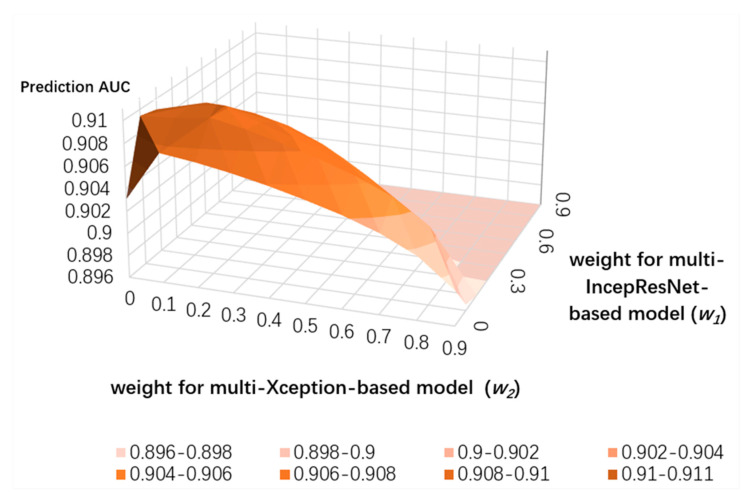
Grid search for optimized weight distribution on ATP-388 over five-fold cross validation.

**Figure 6:**
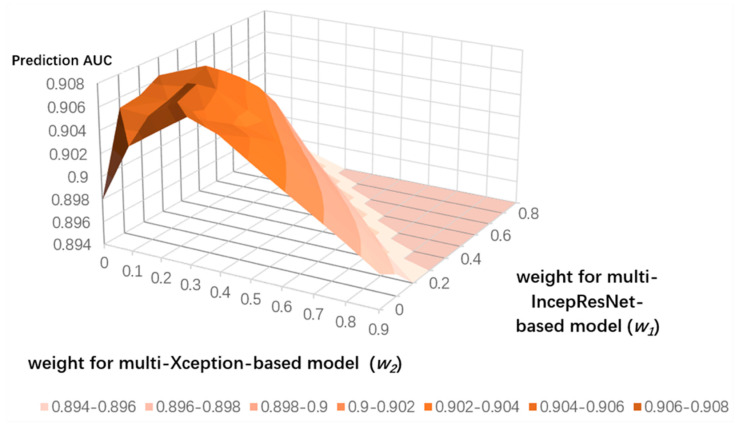
Grid search for optimized weight distribution on ATP-227 over five-fold cross validation.

To search for the optimized weight distribution, we set *w_1_* as the weight for the multi-IncepResNet-based predictor, *w_2_* as the weight for the multi-Xception-based predictor, and the weight of the LightGBM predictor as determined by 1 − *w_1_* − *w_2_*. We conducted a grid search for *w_1_* and *w_2_* from 0.1 to 0.9 with a step size of 0.1 on both ATP-388 and ATP-227 datasets over five-fold cross validation; the results are shown in Figure 5 and Figure 6. Since we needed to represent three variables (weights) on a 2D surface and the sum of variables was equal to 1, when *w_1_* and *w_2_* were set, the corresponding weight for LightGBM was determined by 1 − *w_1_* − *w_2_*. The *x* and *y* axes represent the weights of the multi-IncepResNet-based predictor and multi-Xception-based predictor, respectively, and the *z* axis is the prediction *AUC* given its corresponding weight distribution. The higher the *AUC*, the darker the orange color in the surface diagram. For both ATP-388 and ATP-227, the optimized weight distributions with the *AUC*s of 0.910 and 0.907 were obtained when *w_1_* and *w_2_* were both set to 0.2 and the corresponding weight for the LightGBM predictor was 1 − 0.2 − 0.2 = 0.6. The performance on independent testing sets is reported for this distribution.

## 4. Materials and Methods

### 4.1. Datasets

In this study, two datasets were used: a classic binding dataset and a newly proposed binding dataset. The reason for applying two datasets is that most previously described methods were trained and evaluated on the classic dataset; therefore, to enable comparison between our method and previous methods, the same dataset needed to be used. However, the classic dataset was proposed in 2011, which means that the number of protein sequences in the classic dataset is relatively low. To solve this problem, a newly proposed dataset was also applied in our study to demonstrate the prediction performance of our method on recently curated protein sequences.

#### 4.1.1. ATP-227 and ATP-17

In 2011, Chen et al. [16] developed the ATP-227 dataset, which consists of 227 protein chains with pairwise identities lower than 40% sequence. The binding residue is defined if at least one of its nonhydrogen atoms is less than 3.9 Å away from a nonhydrogen atom of the ATP molecule. The ATP-17 dataset contains those ATP-binding protein chains that were released after March 10, 2010. The maximal pairwise sequence identity in ATP-17 was reduced to 40%. If a given chain shares >40% identity with a chain in ATP-227, then the chain is removed. This process assures that ATP-17 is independent of ATP-227 and can be used as a testing set for models that are trained on ATP-227. As a result, 17 ATP-binding protein chains have been kept in the ATP-17 testing set. The numbers of ATP-binding residues and nonbinding residues in ATP-227 and ATP-17 are listed in Table 7. The ATP-227 and ATP-17 datasets are available at http://biomine.ece.ualberta.ca/ATPsite/.

#### 4.1.2. ATP-388 and ATP-41

In 2018, Hu et al. [19] proposed an ATP-binding dataset that consists of 2144 ATP-binding protein chains, named PATP-2144, which has clear target annotations and was deposited into the Protein Data Bank (PDB) on November 5, 2016. The redundant sequences were then removed using CD-hit [37] software with sequence identity <40%, yielding a total of 429 nonredundant protein sequences. Finally, the 429 nonredundant sequences were divided into a training set (ATP-388) and a testing set (ATP-41). ATP-388 consists of 388 protein chains, which were deposited into the PDB before 5 November 2014, and ATP-41 consists of 41 protein chains that were deposited into the PDB after 5 November 2014. The numbers of ATP-binding residues and nonbinding residues in ATP-388 and ATP-17 are listed in Table 7. The ATP-388 and ATP-41 datasets are available at http://zhanglab.ccmb.med.umich.edu/ATPbind.

### 4.2. Feature Representation

A previous study [39] showed that the binding properties of the target residue are affected by its adjacent residues; therefore, a sliding window was applied in this study to collect the features of both the target residue and its adjacent residues. A sliding window of size L contains the feature of the target residue and the features of (L − 1)/2 adjacent residues on the left and right sides of the target residue. By trialing different sizes of sliding windows, we found that the prediction models achieved better performance when L = 17 than with other sliding window sizes. Therefore, the size of the sliding window was set to 17 in this study.

#### 4.2.1. Position-Specific Scoring Matrix (PSSM)

For a given protein sequence, we generated the PSSM profile by running PSI-BLAST [41] against the Swiss-Prot database with three iterations and an E-value of 10^−3^. The size of PSSM was Lx20, where L represents the length of protein sequence and 20 represents the 20 categories of amino acids. For each value in the profile, we normalized it to the range of 0 and 1 with the following logistic function:

(6) $$S_{i,r}^{\left(N\right)}=\;1/(1+e^{-s_{i,j}}),$$

where $S_{i,r}^{\left(N\right)}$ represents the normalized value and $s_{i,j}$ represents the original value in the PSSM. Finally, for the sliding window with size 17, the dimension of the PSSM feature was 17 × 20 = 340.

#### 4.2.2. Predicted Secondary Structure

A previous study [8] showed that protein secondary structure is relevant to ATP-binding properties. More specifically, the ratios of three secondary structure classes (coil (C), helix (H), and strand (E)) in ATP-binding residues and nonbinding residues are different. In this study, PSIPRED [42] was applied to predict the secondary structure for the query residue based on the sequence information. In the sliding window with size 17, the dimension of predicted secondary structure feature was 17 × 3 = 51.

#### 4.2.3. Residue One-Hot Encoding

According to the dipoles and volumes of side chains of amino acids, 20 categories of amino acids can be divided into seven classes [43] as follows: Class A = {Ala, Gly, Val}, Class B = {Ile, Leu, The, Pro}, Class C = {His, Asn, Gln, Trp}, Class D = {Tyr, Met, Thr, Ser}, Class E = {Arg, Lys}, Class F = {Asp, Glu}, and Class G = {Cys}. Therefore, a seven-dimensional one-hot binary key was used to encode the physicochemical property for each residue. In the sliding window with size 17, there were 17 × 7 = 119 dimensions of one-hot encoding features.

In summary, for each query residue, three types of sequence-based features were extracted: the PSSM feature, the predicted secondary structure feature, and the one-hot encoding feature. The feature matrices for each type of features were 17 × 20, 17 × 3, and 17 × 7, respectively.

### 4.3. Deep Convolutional Neural Network

#### 4.3.1. The Multi-IncepResNet-Based Predictor

In practical applications, using the basic CNN architecture may not produce satisfying performance; therefore, various CNN-based network architectures have been developed, such as VGGnet, InceptionNet, and ResNet. Among these architectures, InceptionNet and ResNet can efficiently overcome the problem of gradient vanishing and maintain stable performance in practical applications [44,45].

The InceptionNet structure applies multiple convolution operations concurrently with different sizes of convolution kernels, which detect local features from various receptive fields. These features are then concatenated for better representation in deeper layers. In the ResNet structure, the shortcut connection propagates the features from one block directly into other blocks in the network. This procedure enables the flow of information across the convolution layers and avoids the attenuation caused by multiple stacked nonlinear transformations. Therefore, by improving the optimization and reducing the number of parameters, the ResNet structure avoids network degradation and overfitting.

In this study, we propose a multi-IncepResNet-based predictor which combines the InceptionNet and ResNet for protein–ATP binding residue prediction. Compared with normal CNN structures that take the whole feature matrix as the input, we feed the involved features individually into the network and extract deep features separately via convolution kernels. Using individual features for convolution may reduce the negative effects caused by the differences between various features. For the PSSM feature and one-hot encoding feature, two stacked Inception blocks are applied for deep information extraction, and the shortcut connection is used to propagate the input feature into the deeper layer of the network and concatenate with the output of Inception blocks. For the predicted secondary structure feature, considering its low dimension, only one Inception block is used. Finally, the deep representations from three individual features are combined and perform classification using the following fully connected layers.

**Figure 7:**
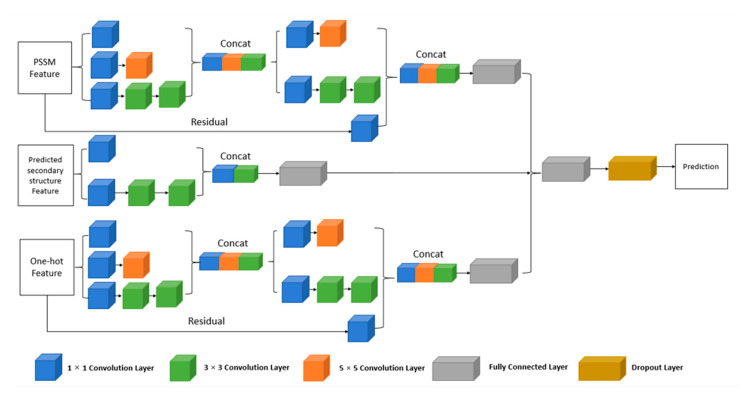
The structure of the proposed multi-IncepResNet-based predictor.

During the training process, a batch-normalization layer was used as a regularizer after any convolution operation to avoid the gradient-vanishing problem. The multi-IncepResNet-based predictor was implemented on the the Keras framework library (version 2.2.4) with a Tensorflow backend. In our experiment, many network parameters and training parameters were tried. For the results reported, the optimizer used for training was Adam, the initial learning rate was set to 10^−4^, the batch size was 256, and the maximum number of epochs was set up to 60. To avoid overfitting, the dropout approach was adopted and the dropout rate was 0.4. The early stopping mechanism from Keras was used to stop network training when the monitored validation loss stopped improving; the patience (i.e., the threshold number of epochs with no improvement after which training is stopped) was set to 8. The structure of the proposed multi-IncepResNet-based predictor is shown in Figure 7.

#### 4.3.2. The Multi-Xception-Based Predictor

According to Chollet et al. [35], the purpose of a typical Inception block is to decouple the cross-channel correlations and spatial correlations. More specifically, a typical Inception block first looks at cross-channel correlations via a set of 1 × 1 convolutions, mapping the input data into three or four separate spaces, and then maps all correlations in these smaller 3D spaces using 3 × 3 or 5 × 5 convolutions. Based on this background, a stronger hypothesis can be formed: the mapping of cross-channel correlations and spatial correlations in the feature maps can be entirely decoupled. Since this hypothesis is the extreme version of an Inception block, the network structure based on this hypothesis is named Xception.

Xception consists of two steps: pointwise convolution and depthwise convolution. In pointwise convolution, a 1 × 1 convolution is used to transform the number of input channels to a new channel depth. In depthwise convolution, each channel of the input is convolved separately and then stacked together. To improve the overall performance, the residual connection is adopted in Xception between different layers.

**Figure 8:**
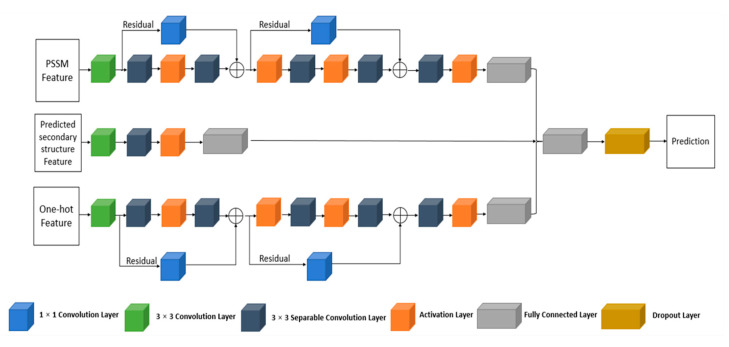
The structure of the proposed multi-Xception-based predictor.

In our proposed multi-Xception-based predictor, the features are separately fed into the corresponding Xception network structures to extract deep representations and reduce feature differences. The PSSM feature and one-hot encoding feature deploy six stacked convolution layers with residual connections for feature extraction; the predicted secondary structure feature deploys two stacked convolution layers without residual connection because of its low dimensionality. The multi-Xception-based predictor was also implemented on TensorFlow and Keras. The settings of hyperparameters were the same as for the Multi-IncepResNet-based predictor. The dropout procedure and early stopping mechanism were also adopted to avoid overfitting. The full structure of the multi-Xception-based predictor is shown in Figure 8.

### 4.4. LightGBM Predictor

Gradient Boosting Decision Tree (GBDT) [46] is an iterative algorithm based on decision tree and can be used for classification and regression. Given a training dataset {*x_1_, x_2_, …, x_n_*}, after each gradient enhancement iteration, a negative gradient {*g_1_, g_2_, …, g_n_*} of the loss function from the model output is obtained. The feature with the largest information gain is then selected in the decision tree to partition each node. The information gain in the GBDT is defined as follows:

(7) $$V_{j|o}\left(d\right)=\;\frac{1}{n_{O}}\left(\frac{{\left(\sum_{\left\{x_{i}\in O:x_{ij}\leq d\right\}}g_{i}\right)}^{2}}{n_{l|O}^{j}\left(d\right)}+\frac{{\left(\sum_{\left\{x_{i}\in O:x_{ij}>d\right\}}g_{i}\right)}^{2}}{n_{r|O}^{j}\left(d\right)}\right),$$

(8) $$n_{O}\;=\;\sum I\left[x_{i}\in O\right],$$

(9) $$n_{l|O}^{j}=\;\sum I\left[x_{i}\in O:x_{ij}\leq d\right],$$

(10) $$n_{r|O}^{j}=\;\sum [x_{i}\in O:x_{ij}>d].$$

The majority of the computational time required by the traditional GBDT algorithm is typically consumed in the construction of a decision tree, which needs to find the optimal segmentation point. The general method involves sorting feature values and enumerating all possible feature points, which wastes considerable time and memory. To solve this problem, the LightGBM algorithm was proposed, which uses an improved histogram algorithm. LightGBM divides the continuous eigenvalues into K intervals and the division point is selected from among the K values. This process greatly speeds up the forecasting speed and reduces memory usage without reducing the prediction accuracy. In addition to the histogram algorithm, LightGBM applies another two techniques to improve the performance and computational efficiency: gradient-based one-side sampling (GOSS) and exclusive feature bundling (EFB). The absolute gradient of each training instance is calculated and sorted in GOSS. The training instances with larger gradient have greater impacts on the information gain. Therefore, the first α×100% instances with larger gradients are selected as a Subset A. The remaining instances with smaller gradients are randomly sampled to obtain Subset B, with an instance size of $b\times \left|A^{c}\right|$. Finally, the information gain is calculated as follows:

(11) $$\bar{V_{j}\left(d\right)}=\;\frac{1}{n}\left(\frac{{\left(\sum_{\left\{x_{i}\in A:x_{ij}\leq d\right\}}g_{i}+\frac{1-a}{b}\sum_{\left\{x_{i}\in B:x_{ij}\leq d\right\}}g_{i}\right)}^{2}}{n_{l}\left(d\right)}+\;\frac{{\left(\sum_{\left\{x_{i}\in A:x_{ij}>d\right\}}g_{i}+\frac{1-a}{b}\sum_{\left\{x_{i}\in B:x_{ij}>d\right\}}g_{i}\right)}^{2}}{n_{r}\left(d\right)}\right).$$

In the EFB method, one of the features is selected as a vertex, and the other features are selected in turn. If the other features are not mutually exclusive with the selected feature, edges are added for each feature. The greedy algorithm is used to produce good bundling characteristics. An offset is then added to the original value of the feature so that the proprietary feature residues can be retained in different bins to construct the feature bundle, and the value of the original function can be identified from the function package. Finally, the features in the same bundle are merged to divide the features into a minimum number of bundles.

### 4.5. Complementary Template-Based Prediction Method

If two protein sequences share similar amino acid compositions and are homologous, the similar segments in their sequences are likely to have common functions [47]. Based on this, we tried to find the homologous sequences for a query protein sequence in the training set in which the ATP-binding residues were clearly annotated. The homologous sequences from the training set can be regarded as the sequence template for the query sequence. In this study, the PSI-BLAST program was applied to search the sequence templates for the query sequence against the training set. The bit score in the output of PSI-BLAST reflects the homology between the query sequence and each sequence in the training set. If the bit score was larger than the threshold, we checked the segments that were similar between the query sequence and the template to determine whether ATP-binding residues existed in these segments. If ATP-binding residues were detected in segments similar to the template sequence, the corresponding residues in the query sequence were predicted as ATP-binding residues. Otherwise, if there was no ATP-binding residue in the similar segment or the template sequence could not be found in the training set, the sequence template-based prediction method was not applied. We trialed various bit-score thresholds and set the threshold to 50, which produced the most accurate performance. Finally, the result of the sequence template-based method was combined with the result from ensemble predictors to form the final prediction. According to the experimental results, the sequence template-based method produced good accuracy for positive samples, which means the ATP-binding residue predictions produced by the template-based method were relatively accurate. However, we also found that the prediction coverage in the results of template-based method was not satisfactory, which means there were many true ATP-binding residues that could not be identified because the number of protein sequences in the training set was very limited, so the method could not find enough templates for prediction. Therefore, in this study, the sequence template-based method was regarded as a complementary method that was used to further improve the performance of ensemble predictors.

### 4.6. Imbalanced Learning Problem

Since ATP is a fairly small molecule, the number of nonbinding residues is much larger than the number of binding residues in ATP-binding protein sequences. Therefore, ATP-binding residue prediction is a typical imbalanced learning problem. In training set ATP-227, the ratio between nonbinding residues and binding residues is about 23.70; in training set ATP-388, the ratio is 25.12. Thus, if any classification algorithm is directly applied to such an imbalanced dataset, the classifier will easily predict every residue as nonbinding. Previous studies [17,19] have often used undersampling or upsampling techniques to construct a relatively balanced dataset, but these procedures inevitably lose some information or pollute the original dataset. In this study, a balanced-learning approach was adopted to handle the problem. For CNN-based predictors, the weighted cross-entropy was used as the loss function in the network, which is defined as follows:

(12) $$H=\sum_{l}W*y_{i}*H\left(i\right)\;,$$

(13) $$\forall i\in \left[0,N\right),\;H\left(i\right)=-\sum_{i}y_{i,l}*\log\hat{y_{i,l}},$$

(14) $$W=\frac{N}{l*bincount\left(y_{i}\right)},$$

where *N* is the total number of data instances, *l* is the number of classes, *W* is the weight for each class, $y_{i}$ is the ground truth label with one-hot encoding, $\hat{y_{i}}$ is the predicted probability from the classifier, *H(i)* is the cross-entropy for one datum instance, and *H* is the weighted cross-entropy. Using the weighted cross-entropy as the loss function, an imbalanced learning problem can be solved by assigning different weights to rescale the prediction of each class.

For the LightGBM classifier, the hyper parameter *scale_pos_weight* can be set to give a larger weight for positive data instances. We used a similar method, which was applied in CNN-based predictors to calculate the weight for positive instances, i.e., Equation (11).

### 4.7. Architecture of Proposed Ensemble Prediction Method

**Figure 9:**
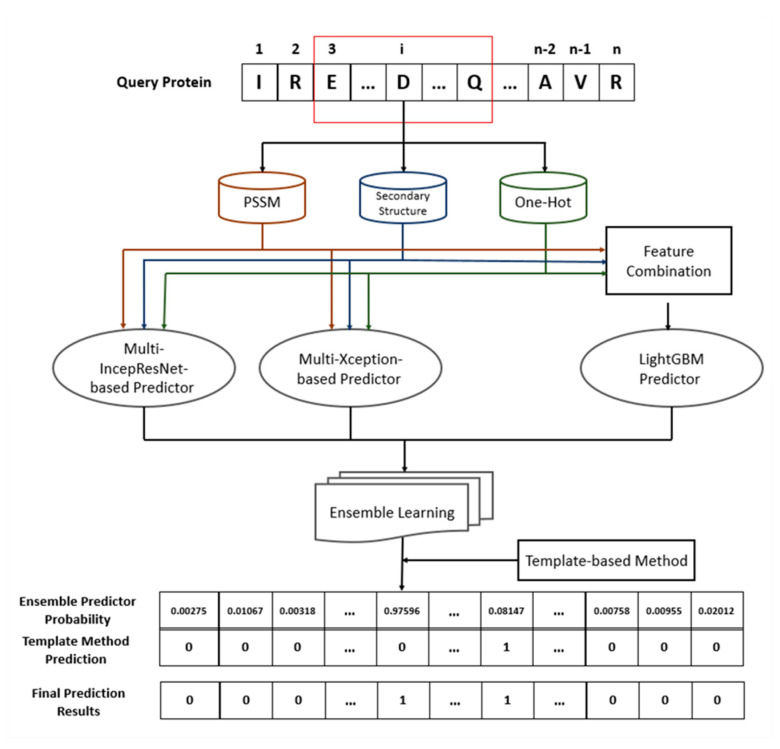
The architecture of the proposed ensemble prediction method.

Figure 9 illustrates the architecture of the proposed ensemble prediction method for protein–ATP binding residues. For a query protein, three types of features are extracted from the protein sequence: the PSSM, the predicted secondary structure, and the one-hot encoding. The three features are then separately sent into the CNN-based classifiers, including the multi-IncepResNet-based predictor and the multi-Xception-based predictor. After the feature combination, the combined feature is sent to the LightGBM predictor. The outputs of three subclassifiers are merged by the ensemble learning algorithm. Finally, complemented with the template-based method, a residue is identified as ATP-binding if its ensemble predictor probability is larger than the prediction threshold, or if it is matched by the template from the training set.

### 4.8. Performance Evaluation

In this study, four routinely used evaluation criteria were applied to examine the overall performance of the proposed method: overall accuracy (*ACC*), sensitivity (*Sen*), specificity (*Spe*), and Matthews correlation coefficient (*MCC*). These evaluation criteria are commonly applied in bioinformatics research [38,40] to reveal classification performance. The definitions of these criteria are as follows:

(15) $$ACC=\;\left(TP+TN\right)/\left(TP+TN+FP+FN\right),$$

(16) $$Sensitivity=\frac{TP}{TP+FN},$$

(17) $$Specificity=\frac{TN}{TN+FP},$$

(18) $$MCC=\frac{TP*TN-FP*FN}{\sqrt{\left(TP+FP\right)\left(TP+FN\right)\left(TN+FP\right)\left(TN+FN\right)}},$$

where *TP*, *TN*, *FP*, and *FN* represent the number of true positive instances, true negative instances, false positive instances, and false negative instances, respectively. Since these evaluation criteria are threshold-dependent, they reflect the prediction performance under a specific threshold. To fairly compare our proposed method with other sequence-based prediction methods, we applied the same procedure as used with other methods [8,17,19,48,49] to determine the prediction threshold, in which the threshold that maximizes the *MCC* value is selected. For a soft-type classifier that outputs a continuous numeric value to represent the probability of an instance belonging to a predicted class, selecting different prediction thresholds produces different corresponding prediction confusion matrices, which leads to fluctuation of threshold-dependent criteria. If severe imbalances exist in the benchmark dataset, threshold-dependent criteria sometimes fail to objectively report the performance, as they are strongly affected by the ratio of positive and negative instances in the dataset. Therefore, the receiver operating characteristic (*ROC*) curve was adopted to reveal the performance of the proposed prediction method. The *x* axis and *y* axis in the *ROC* curve were false rate (*FPR*) and true positive rate (*TPR*), respectively, which can be described as follows:

(19) $$FPR=FP/\left(TN+FP\right),$$

(20) $$TPR=TP/\left(TP+FN\right).$$

The definition of *TPR* is the same as that of sensitivity—it mainly concerns how many true positive instances are correctly predicted—and *FPR* mainly concerns how many true negative instances are incorrectly predicted. Thus, the values of *TPR* and *FPR* do not change when the ratio between the positive and negative instances fluctuates because *TPR* and *FPR* concern the predicted classes (*TPR* for positive instances and *FPR* for negative instances). This is why the *ROC* curve is not greatly affected by an imbalanced distribution in the dataset, and can more objectively evaluate the performance under an imbalanced learning situation. The area under curve (*AUC*) which is totally threshold-independent, can be calculated to reveal the performance of a classification algorithm. A classification algorithm with a larger *AUC* value means the algorithm shows a stronger and more stable prediction performance.

## 5. Conclusions

Using sequence-based computational model to predict ATP-binding residues in proteins is significant for protein function annotation and protein structure detection. Since the number of proteins with known structure is relatively smaller than the number of proteins with known sequence, for a certain protein sequence with no structure information, computational model can accurately predict the ATP-binding residues in the protein sequence which provides guiding significance in function analysis and drug development on this protein. Moreover, when facing with large-scale protein data, using computation model to predict ATP-binding residues is much economical and time-saving compared with biochemical experimental methods.

In this study, we constructed an ensemble-learning-based prediction method for protein–ATP binding residues, which combines a deep convolutional neural network and the LightGBM. The predictor uses the protein sequence information features as features, which include the PSSM, the predicted secondary structure, and the one-hot encoding. Since protein–ATP binding residue prediction is a typical imbalanced learning problem, we distributed the specific weights in the loss functions, which were calculated according to the ratio between the positive instances and the negative instances to solve the imbalance problem. As the core of the prediction method, we developed three subclassifiers: a multi-IncepResNet-based predictor, a multi-Xception-based predictor, and a LightGBM predictor. For each query residue, the classification probability of the ensemble predictor was obtained by combining the probabilities from the three subclassifiers with an optimized weight distribution. To further improve the overall prediction performance, a complementary template prediction method was also adopted. The outstanding performance of our proposed ensemble predictor indicates that using ensemble learning algorithm in combination with a deep convolutional neural network and LightGBM is a useful tool for protein–ATP binding residue prediction. Our work enriches the protein–ATP binding residue prediction ability using sequence information, and the method could be applied to other protein–ligand binding residue prediction problems in future works.

## Figures and Tables

**Table 3:** Hyperparameters used for tuning the 2D convolutional neural network (CNN) model.

Hyperparameter	Value
Optimizer	Adamdelta, Adamgrad, Adam, RMSprop, SGD, Adamax
Number of epochs	30 to 120
Batch size	32, 64, 128, 256
Learning rate	1 × 10^−1^, 1 × 10^−2^, 1 × 10^−3^, 1 × 10^−4^
Dropout rate	0.1, 0.2, 0.3, 0.4, 0.5

**Table 4:** Prediction performance of multi-IncepResNet-based predictor, multi-Xception-based predictor, and the fine-tuned 2D CNN model on ATP-388 over five-fold cross-validations (The highest value of each column in the table is shown in bold).

Method	Accuracy (ACC)	Sensitivity (Sen)	Specificity (Spe)	Matthews Correlation Coefficient (MCC)	AUC
Fine-tuned 2D CNN model	0.950	**0.512**	0.967	0.417	0.871
Multi-IncepResNet-based predictor	0.965	0.489	0.984	0.501	0.886
Multi-Xception-based predictor	**0.967**	0.491	**0.986**	**0.519**	**0.892**

**Table 5:** The optimal hyperparameters for CNN structures with combined feature and CNN structures with separate features after fine-tuning.

Hyperparameter	CNN Structure with Combined Feature	CNN Structure with Separate Features
Optimizer	Adam	Adam
Training epochs	60	60
Initial learning rate	0.001	0.0001
Batch size	128	256
Dropout rate	0.4	0.4

**Table 6:** Prediction performance of maximum ensemble, minimum ensemble, mean ensemble, and weighted ensemble on ATP-388 and ATP-227 over five-fold cross validation (The highest value of each column in the table is shown in bold).

Strategy	ACC	Sen	Spe	MCC	AUC
ATP-388					
maximum	0.963	0.557	0.980	0.523	0.902
minimum	0.965	0.481	**0.982**	0.528	0.892
mean	0.966	0.568	0.981	0.544	0.906
weighted	**0.968**	**0.599**	0.981	**0.549**	**0.910**
ATP-227					
maximum	0.965	0.537	0.983	0.536	0.898
minimum	0.967	**0.529**	0.984	0.544	0.893
mean	0.968	0.483	**0.989**	0.552	0.903
weighted	**0.969**	0.493	**0.989**	**0.556**	**0.907**

**Table 7:** The numbers of ATP-binding residues and nonbinding residues in applied datasets.

Dataset	No. of ATP-Binding Residues	No. of Nonbinding Residues	Ratio ^a^
ATP-227	3393	80,409	23.7
ATP-17	248	6974	28.1
ATP-388	5657	142,086	25.1
ATP-41	681	14,152	20.8

^a^: Ratio = Number of nonbinding residues/number of ATP-binding residues.
